# A robust zeroing neural network and its applications to dynamic complex matrix equation solving and robotic manipulator trajectory tracking

**DOI:** 10.3389/fnbot.2022.1065256

**Published:** 2022-11-15

**Authors:** Jie Jin, Lv Zhao, Lei Chen, Weijie Chen

**Affiliations:** ^1^School of Information Engineering, Changsha Medical University, Changsha, China; ^2^School of Information and Electrical Engineering, Hunan University of Science and Technology, Xiangtan, China

**Keywords:** recurrent neural network, zeroing neural network, dynamic complex matrix equation, activation function, convergence

## Abstract

Dynamic complex matrix equation (DCME) is frequently encountered in the fields of mathematics and industry, and numerous recurrent neural network (RNN) models have been reported to effectively find the solution of DCME in no noise environment. However, noises are unavoidable in reality, and dynamic systems must be affected by noises. Thus, the invention of anti-noise neural network models becomes increasingly important to address this issue. By introducing a new activation function (NAF), a robust zeroing neural network (RZNN) model for solving DCME in noisy-polluted environment is proposed and investigated in this paper. The robustness and convergence of the proposed RZNN model are proved by strict mathematical proof and verified by comparative numerical simulation results. Furthermore, the proposed RZNN model is applied to manipulator trajectory tracking control, and it completes the trajectory tracking task successfully, which further validates its practical applied prospects.

## Introduction

Complex matrix problems frequently arise in mathematics and engineering, since complex matrices are widely applied in signal processing (Liu et al., 2014), image quality assessment (Wang, 2012), joint diagonalization (Maurandi et al., 2013), and robot path tracking (Guo et al., 2019, 2020; Jin et al., 2020, 2022a,c,e; Shi et al., 2021, 2022a; Liu et al., 2022). Various numerical algorithms have been presented to solve the complex matrix problems, such as the Newton iterative method (Rajbenbach et al., 1987) and the Greville recursive method (Gan and Ling, 2008). However, the complexity of these iterative algorithms is proportional to the dimension of the matrix to be calculated, and these iterative algorithms are very effective in the calculation of low dimensional matrix. As the dimension of the matrix increases, the computational workload also increases dramatically. Moreover, with the development of big data science, the demand for large-scale computation is also inevitable. Owing to their serial-processing characteristic, the powerlessness of iterative algorithms in large-scale computation are gradually revealed.

To solve the above mentioned issue, the neural network method is proposed and deeply investigated due to its potential advantages of distributed-storage and parallel-computation in large-scale computation (Lin et al., 2022a,b; Zhou et al., 2022a,b). As a typical recurrent neural network (RNN), the gradient-based neural network (GNN) is widely used to solve matrix problems in recent years (Liu et al., 2021; Jin et al., 2022b). For example, an odd activation function (AF) activated GNN model is presented in Zhang (2005), and it solves matrix inversion problem effectively. Besides, an improved GNN model for solving linear inequalities is presented in Xiao and Zhang (2011). The GNN model can only approach the theoretical solutions of time-varying problems with fluctuation, rather than precisely converging to their theoretical solutions, and they are commonly used to solve static problems. However, time-varying problems are often encountered with the increasingly development engineering techniques, and it is urgent to develop a neural network model for solving time-varying problems.

It is worth to mention that the zeroing neural network (ZNN) model for solving dynamic problems has been proposed by Zhang and Ge (2005). As the time derivative of coefficient matrices is fully considered, the ZNN model achieves accurate solution to dynamic problems, which makes the ZNN model a powerful tool for solving dynamic problems. In Li et al. (2013), a sign-bi-power activation function (SBPAF) activated ZNN model achieves finite-time convergent to the theoretical solution of dynamic linear equation. In Jin (2021b), a finite time convergence recurrent neural network (FTCRNN) model is realized for solving time-varying complex matrix equation, and it has faster convergent speed than the conventional ZNN model. The above mentioned improved ZNN models guarantee accurate and fast solution to dynamic problems in ideal no noise environment. However, noises are unavoidable in reality, anti-noise ability must be considered for all the neural models. Hence, many anti-noise neural models have been reported to address this issue in recent years. In Jin et al. (2016), an anti-noise IEZNN model is reported for dynamic matrix inversion in noise polluted environment. Besides, in Jin et al. (2017), a NTZNN model is presented for solving dynamic problems in noisy environment. The existing anti-noise models work properly in noisy environment, but their finite-time convergent performance can be further improved. Thus, the improvement of the convergence and robustness of the existing neural models is still open. Moreover, the previous neural models focused on solving real domain dynamic problems (Li et al., 2020, 2021, 2022; Gong and Jin, 2021; Jin, 2021a; Jin and Qiu, 2022; Jin et al., 2022d,f; Shi et al., 2022b; Zhu et al., 2022), and the neural network research for solving complex domain dynamic problems is also indispensable. With the expansion of neural models to complex domain, various complex domain scientific and engineering problems can be solved easily.

Inspired by the above mentioned issues, a robust zeroing neural network (RZNN) model with fast convergence and robustness to noises for solving dynamic complex matrix equation (DCME) problems is proposed in this work. Its fast convergence irrelevant to system initial state and robustness to various noises are verified by rigorous mathematical analysis. Besides, the ZNN model activated by SBPAF are also applied to solve the DCME in same condition for the purpose of comparison, and the corresponding simulation results further demonstrate the superior convergence and robustness of the proposed RZNN model for solving dynamic complex domain problems.

## The dynamic complex matrix equation and its transformation

Generally, DCME problem can be described by the following equation.


(1) $$A\,\left(t\right)\,D\,\left(t\right)=B\,\left(t\right)\in {\mathbb{C}}^{n\times \text{n}}$$

where *A(t)* ∈*C^n^*^×^*^n^* and *B(t)* ∈*C^n^*^×^*^n^* are the known dynamic complex matrices, and *D(t)*∈*C^n^*^×^*^n^* represents the unknown dynamic complex matrix to be solved.

As we know, it is very difficult to find the matrix *D(t)* directly from the above complex domain equation. However, any complex number contains real and imaginary parts, and we can solve the complex matrix *D(t)* through the transformation below.


(2) $$\left[A_{\text{re}}\,\left(t\right)+j\,A_{i\,m}\,\left(t\right)\right]\,\left[D_{\text{re}}\,\left(t\right)+j\,D_{i\,m}\,\left(t\right)\right]=B_{r\,e}\,\left(t\right)+j\,B_{i\,m}\,\left(t\right)$$

Then, calculating Eq. 2 yields


(3) $$\left\{ \begin{array}{c} A_{\text{re}}\,\left(t\right)\,D_{\text{re}}\,\left(t\right)-A_{i\,m}\,\left(t\right)\,D_{i\,m}\,\left(t\right)=B_{r\,e}\,\left(t\right)\\ A_{\text{re}}\,\left(t\right)\,D_{i\,m}\,\left(t\right)+A_{i\,m}\,\left(t\right)\,D_{\text{re}}\,\left(t\right)=B_{i\,m}\,\left(t\right) \end{array}\right.$$

Then, Eq. 3 can be simplified as,


(4) $$\left[\begin{array}{c} \begin{array}{cc} A_{\text{re}}\,\left(t\right) & -A_{i\,m}\,\left(t\right) \end{array}\\ \begin{array}{cc} A_{\text{im}}\,\left(t\right) & A_{r\,e}\,\left(t\right) \end{array} \end{array}\right]\,\left[\begin{array}{c} D_{\text{re}}\,\left(t\right)\\ D_{i\,m}\,\left(t\right) \end{array}\right]=\left[\begin{array}{c} B_{\text{re}}\,\left(t\right)\\ B_{i\,m}\,\left(t\right) \end{array}\right]\in {\mathbb{R}}^{2\,\text{n}\times \text{n}}$$

Equation 4 can be further rewritten as,


(5) G⁢(t)⁢X⁢(t)=H⁢(t)

where, $G(t)=\left[\begin{array}{l} \begin{array}{cc} A_{\text{re}}(t) & -A_{im}(t) \end{array}\\ \begin{array}{cc} A_{\text{im}}(t) & A_{re}(t) \end{array} \end{array}\right]\in {\mathbb{R}}^{2n\times 2n}$, $X(t)=\left[\begin{array}{l} D_{\text{re}}(t)\\ D_{im}(t) \end{array}\right]\in {\mathbb{R}}^{2n\times n}$, and $H(t)\text{=}\left[\begin{array}{l} B_{\text{re}}(t)\\ B_{im}(t) \end{array}\right]\in {\mathbb{R}}^{2\text{n}\times \text{n}}$, and we assume det⁡|*D*(*t*)|≠0 to guarantee unique solution of Eq. 5 for *t*∈ [*0*, ∞].

Based on the above transformation, we can know that solving DCME (1) is equivalent to find the solution of the real domain dynamic matrix equation (DME) in (5), and the solution of Eq. 5 satisfies *X(t)* = *D_*re*_(t)* + *jD_*im*_(t)*.

## Zeroing neural network and robust zeroing neural network models

We can follow the steps below to construct the ZNN model for solving DME (5).

Firstly, we define a dynamic error matrix


(6) E⁢(t)=G⁢(t)⁢X⁢(t)-H⁢(t)

Then, formula (7) is applied for the convergence of *E(t)*.


(7) $$\frac{d\,E\,\left(t\right)}{d\,t}=-\gamma \,\Gamma \,\left(E\,\left(t\right)\right)$$

where γ > *0* is an adjustable coefficient for the convergent speed, and Γ*()* is an AF array.

Combining (6) and (7), the ZNN model for solving DME (5) is obtained.


(8) $$G(t)\overset{•}{X}(t)=-\gamma \Gamma \left(G(t)X(t)-H(t)\right)-\overset{•}{G}(t)X(t)+\overset{•}{H}(t)$$

It is worth to point out that the ZNN model (8) is stable as long as AF Γ*()* is a monotonically odd function. As a vital part of the ZNN model, the AF Γ*()* has an important influence on the convergence and robustness of the ZNN model, and various AFs have been reported in recent years, such as the linear AF, bi-power activation function, and SBPAF, etc. In addition, the SBPAF will be adopted as the AF Γ*()* in the ZNN model (8) for the comparisons with the proposed RZNN model in the simulation section.

To further improve the convergence and robustness of the ZNN model, a new AF is presented below.


(9) $$\varphi \,\left(x\right)={\left(a\,{\left|x\right|}^{m}+b\right)}^{k}\,s\,g\,n\,\left(x\right)+c\,x+d\,s\,g\,n\,\left(x\right)$$

where sgn() is the signum function and *m, k, a, b* > *0*, *mk* > *1*.

On the basis of the new AF (9), the RZNN model proposed in this work is realized.


(10) $$G(t)\overset{•}{X}(t)=-\gamma \text{Φ}\left(G(t)X(t)-H(t)\right)-\overset{•}{G}(t)X(t)+\overset{•}{H}(t)$$

where φ(•) is the corresponding element of the AF array Φ(•).

Considering the noises, the noise polluted RZNN model is represented as,


(11) $$G(t)\overset{•}{X}(t)=-\gamma \text{Φ}\left(G(t)X(t)-H(t)\right)-\overset{•}{G}(t)X(t)+\overset{•}{H}(t)+N(t)$$

where *N(t)* denotes the additive matrix noise, and *n_*ij*_(t)* represents its *ij*th element.

## Robust zeroing neural network model analysis

In this section, the convergence and robustness of the proposed RZNN model will be analyzed and verified. For the convenience of subsequent analysis, the following Lemma 1 is introduced in advance.

**Lemma 1.** Consider the following dynamic system (Aouiti and Miaadi, 2020)


(12) $$\overset{•}{x}(t)\leq -{\left(ax^{m}(t)+b\right)}^{k}$$

where *m, k, a, b* > *0*, *mk* > *1*. Dynamic system (12) is fixed-time stable, and *x(t)* will converge to zero within *t*.


(13) t≤1bk(ba)1m(1+1mk−1)

On the basis of Lemma 1, the convergence and robustness of the proposed RZNN model with noise and without noise will be analyzed, respectively.

**Case 1: Without noise**

The following theorem 1 guarantees the fixed-time convergence of the proposed RZNN model (10) in no noise environment.

**Theorem 1.** For arbitrary initial system state, state solution *X(t)* generated by RZNN model (10) will converge to the theoretical solution X*(t) of DME (5) within *t*_*s*_.


ts≤1λbk(ba)1m(1+1mk−1)

Proof. According to (7), the design formula of RZNN model (10) can be derived as $\frac{d\,E\,\left(t\right)}{d\,t}=-\lambda \,\Phi \,\left(E\,\left(t\right)\right)$, and its *n* × *n* subsystems can be presented as


(14) $$\frac{d\,e_{i\,j}\,\left(t\right)}{d\,t}=-\lambda \,\varphi \,\left(e_{i\,j}\,\left(t\right)\right)$$

Consider the Lyapunov candidate function *v_*ij*_(t)* = | *e_*ij*_(t)*|, and substitute AF (9) into (14), then the derivative of *v_*ij*_(t)* is


(15) $$\frac{d\,v_{i\,j}\,\left(t\right)}{d\,t}=\overset{•}{\underset{i\,j}{e}}\left(t\right)\,s\,g\,n\,\left(e_{i\,j}\,\left(t\right)\right)=-\lambda \,\varphi \,\left(e_{i\,j}\,\left(t\right)\right)$$

$$s\,g\,n\,\left(e_{i\,j}\,\left(t\right)\right)=-\lambda \,\left({\left(a\,{\left|e_{i\,j}\,\left(t\right)\right|}^{m}+b\right)}^{k}\,s\,g\,n\,\left(e_{i\,j}\,\left(t\right)\right)+c\,e_{i\,j}\,\left(t\right)+d\,s\,g\,n\,\left(e_{i\,j}\,\left(t\right)\right)\right)$$

(16) $$s\,g\,n\,\left(e_{i\,j}\,\left(t\right)\right)=-\lambda \,\left({\left(a\,{\left|e_{i\,j}\,\left(t\right)\right|}^{m}+b\right)}^{k}+c\,\left|e_{i\,j}\,\left(t\right)\right|+d\right)$$

(17) ≤−λ(a|eij(t)|m+b)k=−(λ1k(avm(t)+b))k

Based on Lemma 1, the convergent time *t*_*ij*_ of the *ij*th subsystem is


tij≤1λbk(ba)1m(1+1mk−1)

Then upper bound of the convergent time of RZNN model (10) is obtained.


ts=max(tij)≤1λbk(ba)1m(1+1mk−1)

**Case 2: Polluted by dynamic bounded disappearing noise (DBDN)**

The following theorem 2 guarantees the fixed-time convergence of the proposed RZNN model (11) polluted by DBDN.

**Theorem 2.** If *N(t)* in (11) is a DBDN, and | *n_*ij*_(t)*| ≤ δ| *e_*ij*_(t)*| and λ*c* ≥ δ [δ∈ (*0*, +∞)] hold. For arbitrary initial system state, state solution *X(t)* generated by RZNN model (11) will converge to the theoretical solution X*(t) of DME (5) within *t*_*s*_.


ts≤1λbk(ba)1m(1+1mk−1)

**Proof.** The evolution formula of RZNN model (11) with noise can be written in the form of $\frac{d\,E\,\left(t\right)}{d\,t}=-\lambda \,\Phi \,\left(E\,\left(t\right)\right)+N\,\left(t\right)$, and its *n* × *n* subsystem can be obtained as


(18) $$\frac{de_{ij}(t)}{dt}=-\text{λ}\varphi (e_{ij}(t))+n_{ij}(t)$$

Consider the Lyapunov candidate function *v_*ij*_(t)* = | *e_*ij*_(t)*| and the inequalities | *n_*ij*_(t)*| ≤ δ| *e_*ij*_(t)*| and λ*d* ≥ δ, substitute AF (9) into (16), then the derivative of *v_*ij*_(t)* is


(19) dvij(t)dt=e•ij(t)sgn(eij(t))=(−λφ(eij(t))+nij(t))sgn(eij(t))=(−λ((a|eij(t)|m+b)ksgn(eij(t))+ceij(t)+dsgn(eij(t)))+nij(t))sgn(eij(t))=−λ(a|eij(t)|m+b)k−λd1|eij(t)|−λd2+nij(t)sgn(eij(t))≤−λ(a|eij(t)|m+b)k+(δ|eij(t)|−λd1|eij(t)|)≤−λ(a|eij(t)|m+b)k=−(λ1k(avm(t)+b))k

Based on Lemma 1, the convergent time *t*_*ij*_ of the *ij*th subsystem is


tij≤1λbk(ba)1m(1+1mk−1)

Then upper bound of the convergent time of RZNN model (11) polluted by DBDN is obtained.


ts=max(tij)≤1λbk(ba)1m(1+1mk−1)

**Case 3: Polluted by dynamic bounded non-disappearing noise (DBNDN)**

The following theorem 3 guarantees the fixed-time convergence of the proposed RZNN model (11) polluted by DBNDN.

**Theorem 3.** If *N(t)* in (11) is a DBNDN, | *n_*ij*_(t)*| ≤ δ and λ*d* ≥ δ [δ∈ (*0*, + ∞)] hold. For arbitrary initial system state, state solution *X(t)* generated by RZNN model (11) will converge to the theoretical solution X*(t) of DME (5) within *t*_*s*_.


ts≤1λbk(ba)1m(1+1mk−1)

**Proof.** The evolution formula of RZNN model (11) with noise can be written in the form of $\frac{d\,E\,\left(t\right)}{d\,t}=-\lambda \,\Phi \,\left(E\,\left(t\right)\right)+N\,\left(t\right)$, and its *n* × *n* subsystem can be obtained as


(20) $$\frac{de_{ij}(t)}{dt}=-\text{λ}\varphi (e_{ij}(t))+n_{ij}(t)$$

Consider the Lyapunov candidate function *v_*ij*_(t)* = | *e_*ij*_(t)*| and the inequalities | *n_*ij*_(t)*| ≤ δ and λ*d* ≥ δ, substitute AF (9) into (18), then the derivative of *v_*ij*_(t)* is


dvij(t)dt=e•ij(t)sgn(eij(t))=(−λφ(eij(t))+nij(t))sgn(eij(t))=(−λ((a|eij(t)|m+b)ksgn(eij(t))+ceij(t)+dsgn(eij(t)))+nij(t))sgn(eij(t))=−λ(a|eij(t)|m+b)k−λc|eij(t)|−λd+nij(t)sgn(eij(t))≤−λ(a|eij(t)|m+b)k+(δ−λd)≤−λ(a|eij(t)|m+b)k=−(λ1n(avm(t)+b))k

According to Lemma 1, the convergent time *t*_*ij*_ of the *ij*th subsystem is


tij≤1λbk(ba)1m(1+1mk−1)

Then, upper bound of the convergent time of RZNN model (11) polluted by DBNDN is obtained.


ts=max(tij)≤1λbk(ba)1m(1+1mk−1)

Based on the above theorems, we can draw the conclusion that the proposed RZNN model can converge to the theoretical solution of DME (5) within fixed-time and it is robust to noise.

## Numerical verification and robotic manipulator application

The convergence and robustness of the proposed RZNN model in noisy environment are analyzed in the above section, two examples of the proposed RZNN for DCME (1) solving and robotic manipulator trajectory tracking will be presented in this section.

**Example 1. DCME (1) solving**

Consider DCME (1) with the following dynamic coefficient matrices.


$$\begin{array}{c} A\,\left(t\right)=\left(\begin{array}{cc} \sin3\,t+j\,\cos3\,t & \sin3\,t-j\,\cos3\,t \end{array}\right)\\ B\,\left(t\right)=\left(\begin{array}{cc} \sin3\,t-j\,\cos2\,t & \left(\cos2\,t+1\right)+j\,\left(\sin3\,t+2\right) \end{array}\right) \end{array}$$

According to the transformation theory in Section “The dynamic complex matrix equation and its transformation,” the above DCME (1) can be transformed to DME (5) with the following dynamic coefficient matrices.


$$G\,\left(t\right)=\left(\begin{array}{c} \begin{array}{cccc} \sin3\,t & \sin3\,t & -\cos3\,t & \cos3\,t \end{array}\\ \begin{array}{cccc} \cos3\,t & -\cos3\,t & \sin3\,t & \sin3\,t \end{array} \end{array}\right)$$

$$\cdot H\left(t\right)=\left(\begin{array}{cc} \sin3\,t & \cos2\,t+1\\ -\cos2\,t & \sin3\,t+2 \end{array}\right)$$

**FIGURE 1:**
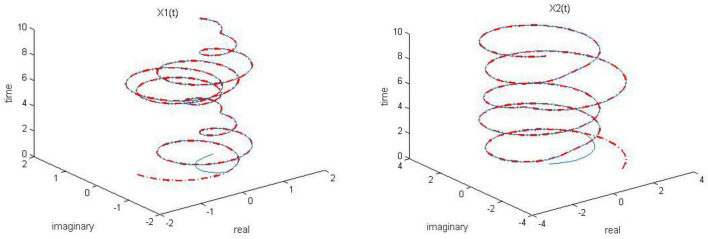
Dynamic complex matrix equation (DCME) (1) solved by robust zeroing neural network (RZNN) model (10) with *k* = 0.5 in no noise environment.

**FIGURE 2:**
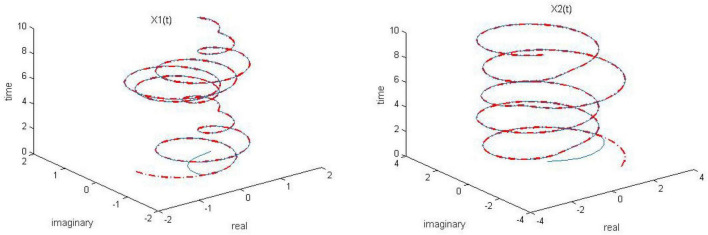
Dynamic complex matrix equation (DCME) (1) solved by robust zeroing neural network (RZNN) model (10) with *k* = 1 in no noise environment.

**FIGURE 3:**
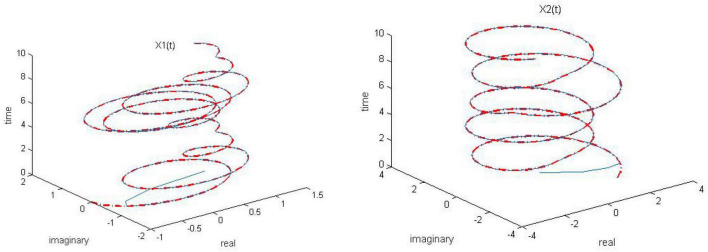
Dynamic complex matrix equation (DCME) (1) solved by robust zeroing neural network (RZNN) model (10) with *k* = 2 in no noise environment.

**FIGURE 4:**
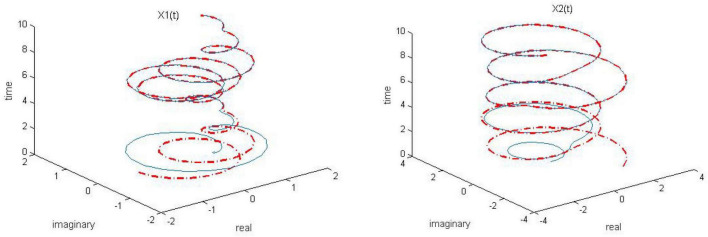
Dynamic complex matrix equation (DCME) (1) solved by sign-bi-power activation function (SBPAF)-based zeroing neural network (ZNN) model (8) in no noise environment.

**FIGURE 5:**
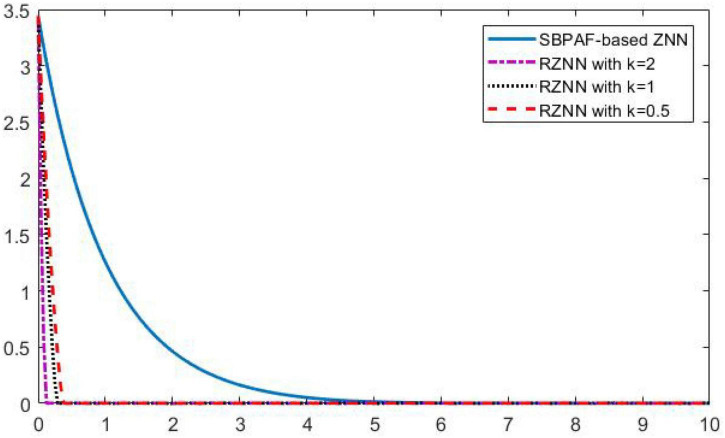
Residual errors of robust zeroing neural network (RZNN) model (10) and sign-bi-power activation function (SBPAF)-based ZNN model (8) in no noise environment.

Let γ = 1, both of the proposed RZNN model (10) and SBPAF-based ZNN model (8) are used to solve the above DME (5) in no noise environment for arbitrary initial state *X(t* = 0). Moreover, in order to observe the parameter *n* in AF (9) to adjust the convergent speed of the proposed RZNN model (10), the parameter *k* is, respectively, set to be *k* = 0.5, *k* = 1, and *k* = 2 for solving DME (5). The corresponding simulation results are presented in Figures 1–5, and solid blue curves are state solutions of DME (5) obtained by neural network models, and red dotted curves are theoretical solutions of DME (5).

As observed in Figures 1–4, both of the proposed RZNN model (10) and SBPAF-based ZNN model (8) effectively solve DME (5) in no noise environment. Moreover, the parameter *k* in AF (9) has an important influence on the convergence of the RZNN model (10), and the convergence of the RZNN model (10) increases with the increase of the parameter *k*. The residual errors | | *G(t)X(t)-H(t)*| | _*F*_ of RZNN model (10) and SBPAF-based ZNN model (8) are presented in Figure 5. From Figure 5, we can clearly observe that the proposed RZNN model (10) has superior convergence than the SBPAF-based ZNN model (8) in no noise environment.

**FIGURE 6:**
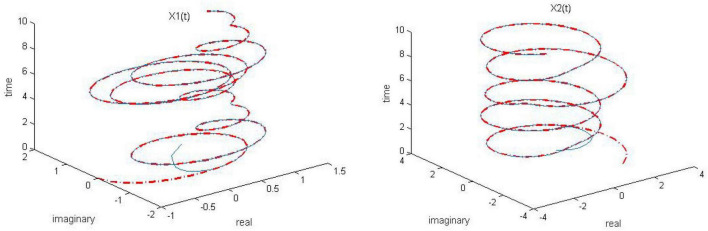
Dynamic complex matrix equation (DCME) (1) solved by robust zeroing neural network (RZNN) model (11) with *k* = 0.5 in *n(t)* = 0.5 polluted environment.

**FIGURE 7:**
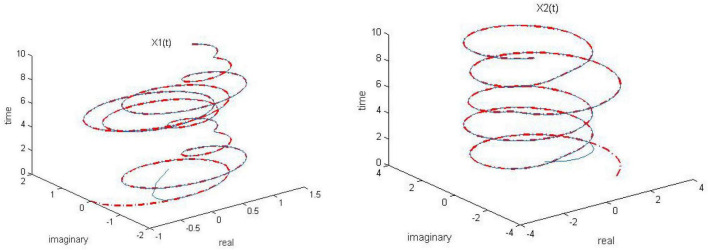
Dynamic complex matrix equation (DCME) (1) solved by robust zeroing neural network (RZNN) model (11) with *k* = 1 in *n(t)* = 0.5 polluted environment.

**FIGURE 8:**
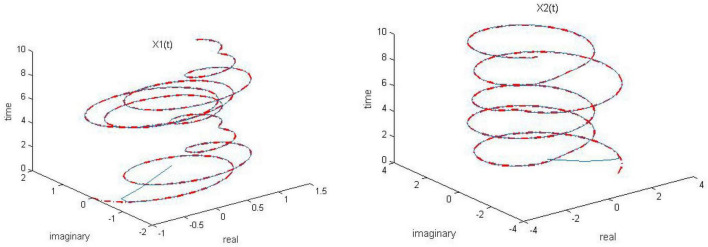
Dynamic complex matrix equation (DCME) (1) solved by robust zeroing neural network (RZNN) model (11) with *k* = 2 in *n(t)* = 0.5 polluted environment.

**FIGURE 9:**
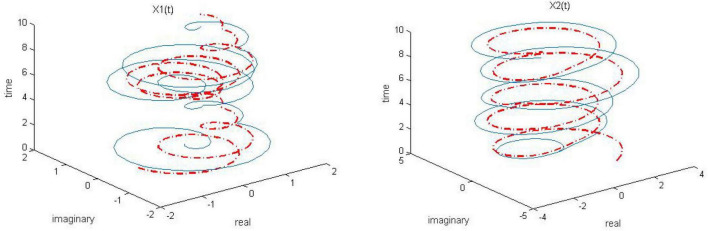
Dynamic complex matrix equation (DCME) (1) solved by sign-bi-power activation function (SBPAF)-based robust zeroing neural network (RZNN) model (8) in *n(t)* = 0.5 polluted environment.

**FIGURE 10:**
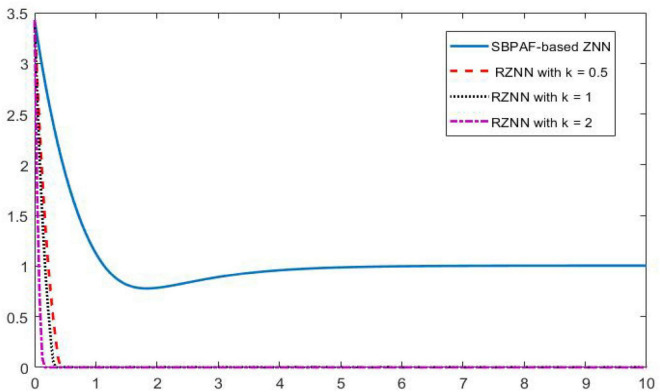
Residual errors of robust zeroing neural network (RZNN) model (11) and sign-bi-power activation function (SBPAF)-based ZNN model (8) in *n(t)* = 0.5 polluted environment.

To further observe the convergence and robustness of the proposed RZNN model (11) and the SBPAF-based ZNN model (8), both of the proposed RZNN model (10) and SBPAF-based ZNN model (8) are adopted to solve the same DME (5) in constant noise *n(t)* = 0.5 polluted environment, and the parameter *n* is also set to be *k* = 0.5, *k* = 1, and *k* = 2, respectively. The corresponding simulation results for solving DME (5) in constant noise *n(t)* = 0.5 polluted environment are presented in Figures 6–10.

As seen in Figures 6–8, the proposed RZNN model (10) still effectively solves DME (5) in noise polluted environment. However, the SBPAF-based ZNN model (8) fails, and it cannot converge to the theoretical solution of DME (5) owing to the influence of the additive noise. The residual errors | | *G(t)X(t)-H(t)*| | _*F*_ of the two models are presented in Figure 10 to further demonstrate their convergence and robustness. From Figure 10, we can also clearly observe that the proposed RZNN model (10) has superior convergence and robustness than the SBPAF-based ZNN model (8) in noise polluted environment.

**Example 2. Robotic manipulator trajectory tracking**

With the development of artificial intelligence, robots have drawn considerable interests in academic and industrial fields (Guo et al., 2021; Jin and Gong, 2021). In this section, a robotic manipulator trajectory tracking application using the proposed RZNN model in noisy environment is presented.

According to Zhou et al. (2022c), the kinematic model of a robotic manipulator is


(21) r⁢(t)=ξ⁢(θ⁢(t))

where *r(t)* is the end-effector position, θ*(t)* is joint angle, *ξ*() stands for a non-linear function. The velocity level motion equation can be expressed as


(22) $$\overset{•}{r}\left(t\right)=J\,\left(\theta \right)\,\overset{•}{\theta }\left(t\right)$$

where *J*(θ) = *əξ*(θ)/*ə*θ.

Assume *r_*d*_(t)* is the desired path, and *r(t)* is the end-effector tracking trajectory. We will design a control law $\mu =\overset{•}{\theta }$, which enforces the tracking error *e(t)* = r(t)–r_*d*_(t) converging to 0. To achieve such a purpose, the proposed RZNN model is used to design the control law, and the RZNN-based kinematic control model is shown below.


(23) J⁢μ•=-J•θ+rd••d-λ⁢Φ⁢(J⁢μ-rd•d)+n⁢(t)

**FIGURE 11:**
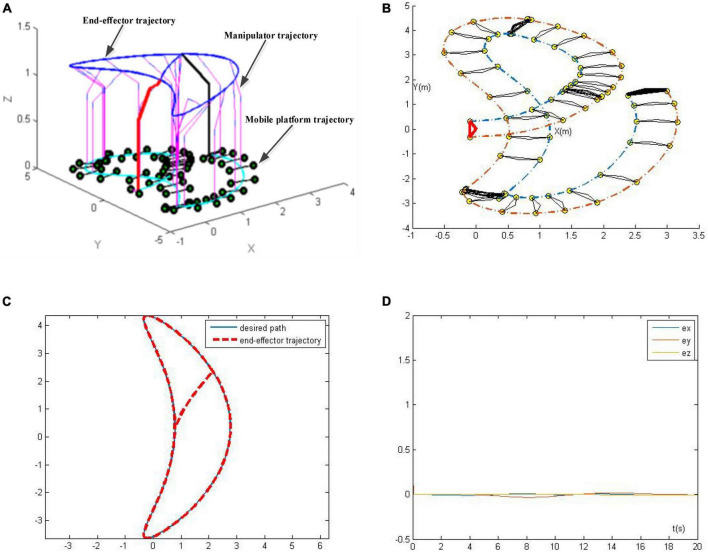
Trajectory tracking results of the robotic manipulator synthesized by robust zeroing neural network (RZNN) model with *n(t)* = 0.5. **(A)** Whole tracking trajectory of the manipulator, **(B)** mobile platform trajectory, **(C)** desired path and the end-effector trajectory, and **(D)** tracking errors.

The corresponding simulation results are presented in Figure 11. Figure 11A is the overall view of the tracking trajectory, Figure 11B is the mobile platform trajectory, Figure 11C presents the actual trajectory of end-effector and the desired tracking path, and Figure 11D presents tracking errors of the robotic manipulator in X, Y, and Z directions. As seen in Figure 11, the RZNN-based kinematic control model (21) completes the trajectory tracking task successfully, and the tracking errors of the robotic manipulator in X, Y, and Z directions are all less than 0.1 mm in noise polluted environment, which further demonstrates its superior convergence and robustness to noise.

## Conclusion

In this paper, by introducing a new AF, a RZNN model for DCME solving and robotic manipulator trajectory tracking is presented. Rigorous mathematical verification demonstrates that the RZNN model can accurately and quickly solve the DCME problem in various noises polluted environment. Moreover, the convergence and robustness of the proposed RZNN model are verified by comparative numerical simulation results. Compared with the SBPAF-based ZNN model, the proposed RZNN model has superior convergence and robustness to noise. In addition, we could focus our future research directions on the further improvements of the convergence and robustness of the RZNN model and the engineering application expansion of the ZNN models.

## Data availability statement

The original contributions presented in this study are included in the article/supplementary material, further inquiries can be directed to the corresponding author.

## Author contributions

All authors listed have made a substantial, direct, and intellectual contribution to the work, and approved it for publication.
